# Cyclic Dipeptides Mediating Quorum Sensing and Their Biological Effects in *Hypsizygus Marmoreus*

**DOI:** 10.3390/biom10020298

**Published:** 2020-02-13

**Authors:** Shu-Jing Sun, Yun-Chao Liu, Cai-Hong Weng, Shi-Wei Sun, Fan Li, Hui Li, Hu Zhu

**Affiliations:** 1 College of Life Sciences, Fujian Agriculture and Forestry University, Fuzhou 350002, China; 2 Centre for Bioengineering and Biotechnology, China University of Petroleum (East China), 66 Changjiang West Road, Qingdao 266580, Chinasunshiwei_1986@163.com (S.-W.S.); lihui@upc.edu.cn (H.L.); 3 Fujian Provincial University Engineering Research Center of Industrial Biocatalysis, College of Chemistry and Materials Science, Fujian Normal University, 32 Shangsan Road, Fuzhou 350007, China

**Keywords:** *Serratia odorifera*, quorum sensing, diketopiperazines, *Hypsizygus marmoreus*, growth and development

## Abstract

A novel quorum sensing (QS) system was discovered in *Serratia odorifera*, the symbiotic bacterium of *Hypsizygus marmoreus*. This system uses cyclo(Pro-Phe), cyclo(Pro-Tyr), cyclo(Pro-Val), cyclo(Pro-Leu), cyclo(Tyr-Leu), and cyclo(Tyr-Ile) as autoinducers. This discovery is the first attempt to characterize cyclic dipeptides as QS signaling molecules in *S. odorifera* and improves the classical QS theory. Significantly, *except for* cyclo(Tyr-Leu), these QS autoinducers can increase the transcription level of lignin-degrading enzyme genes of *H. marmoreus*. The cyclo(Pro-Phe) can increase the activity of extracellular laccase (1.32-fold) and manganese peroxidase (*20%*), which may explain why QS potentially regulates the hyphal growth, primordium formation, and fruit body development of *H. marmoreus*. Furthermore, it was demonstrated that the cyclo(Tyr-Ile) biosynthesis in *S. odorifera* was catalyzed by the nonribosomal peptide synthetase (NRPS). This study supports exploring the growth and development of *H. marmoreus* promoted by its symbiotic bacteria at QS signal transduction level.

## 1. Introduction

Quorum sensing (QS) is a unique way of communication between bacteria and gene regulation systems relying on population density [1]. This signaling transduction mechanism can induce or inhibit the expression of a series of downstream genes to regulate a variety of physiological processes [2], including bioluminescence [3], biofilm formation [4], extracellular polysaccharide synthesis [5], antibiotic production [6], population mobility [7], and the symbiosis of bacteria and eukaryotes [8]. Most QS signaling molecules produced by Gram-negative and Gram-positive bacteria are *N*-acylhomoserine lactones (AHLs) and modified oligopeptides (also called autoinducing peptides, AIPs), which have been widely studied as a response in bacterial QS systems [9]. Interestingly, a furanosyl borate diester (AI-2) that was discovered in *Vibrio harveyi* can function as a kind of QS signaling molecule and a language and participate in signal transduction in different bacterial species. AI-2 is synthesized and catalyzed by the Pfs and LuxS pathway through the activated methyl cycle (AMC) to form a toxic metabolic intermediate, S-acyl homocysteine (SAH), in cells [10]. The PFS enzyme rapidly hydrolyzes SAH to S-ribose homocysteine (SRH) and adenine, and then LuxS converts SRH to 4,5-Dihydroxy-2,3-pentanedione (DPD) and homocysteine acid. The unstable molecule DPD spontaneously cyclizes to form pro-AI-2, and the active signal molecule AI-2 is synthesized with the addition of borate [11]. In addition, indole and its derivatives, generally produced by plants and soil-associated bacteria, are regarded as potential signaling molecules that are generated from the amino acid tryptophan by tryptophanase encoded by the *tnaA* gene [12]. This type of signaling molecule affects the QS-regulated phenotypes (pathogenicity, virulence factor production, and biofilm formation) of *Pseudomonas aeruginosa* [13]. Diketopiperazines (DKPs) are cyclic dipeptides and their derivatives, which contain a stable six-membered ring structure. Recently, related studies showed that natural DKPs have important and diverse biological activities, such as antibacterial, antifungal, antiviral, and antitumor activities [14,15,16]. It is noteworthy that some DKPs can form novel QS signal molecules involved in intercellular communication. At present, reactions in most biosynthetic pathways of DKPs discovered in *Streptomyces* are catalyzed by NRPSs, which can stimulate receptor proteins and activate the transcription of target genes [17].

*H. marmoreus*, one of the most popular edible and medicinal fungi widely cultivated in East Asia, is not only rich in nutritious ingredients and great flavor, but also has a high medicinal value, which therefore made it a large-scale factory-cultivated fungus [18]. *H. marmoreus* contains many biologically active ingredients, especially compounds extracted from its fruit bodies, which exert a variety of prominent pharmacological effects, including antitumor, antioxidation, and antimalignant cell proliferation effects [19,20]. Although *H. marmoreus* has been cultivated in China, the industrial development of its large-scale cultivation is greatly limited because of low productivity, strict cultivation conditions, and low antipathogen activity. In recent years, it has been reported that the bacterial QS system can regulate the growth and development of plants and microorganisms [21]. Different bacteria can substantially enhance crop yields by QS-mediated interaction between plants and microorganisms [22], and various types of signaling molecules can also increase disease resistance in plants. Currently, the specific mechanism of this action is not yet clear. The earliest study of this relationship between bacterial QS signal molecules and physiological activities of eukaryotic organisms was derived from Mathesius’s research, which demonstrated that AHLs could activate the growth factor GH3 promoter and upregulate auxin-related genes [23]. Some other experiments [24] also indicated that *N*-hexanoyl-homoserine lactone (C_6_-HSL) and *N*-Octanoyl-L-homoserine lactone (C_8_-HSL) signaling molecules were beneficial to the growth and metabolism of roots from *Arabidopsis thaliana*. The 3-oxo-C_14_-HSL produced by *Sinorhizobium meliloti* can effectively enhance the nodulation of roots from *Medicago truncatula* [25]. Other QS signaling molecules have no such promoting effects. Bacterial interaction with eukaryotes is a two-way exchange of information mediated by the QS system. Although this symbiotic mechanism is still unknown, this interaction between bacterial QS signaling molecules and eukaryotic host cells creates new possibilities for in-depth investigation of the growth and development of eukaryotic organisms from the constructivist point of view. In nature, bacteria often colonize fungi [26], which indicates that the bacterial QS system affects the morphological structure and physiological functions of fungi. The detailed study on the mechanism of beneficial microorganisms promoting the growth and development of edible fungi via QS system has drawn much attention. It is of great practical significance for shortening the edible fungi growth cycle, increasing yield, and enhancing economic benefits.

At present, most studies on *H. marmoreus* focus on the breeding of new high-yield varieties, the selection and optimization of culture conditions, and active ingredient analysis [20]. At the same time, there has been little research reported on the interaction between coexisting microorganisms and the growth and development of *H. marmoreus*. In our former research, 34 bacterial phyla were detected in cultivation bags by Illumina HiSeq 2500 platform. Serratia species increased exponentially reaching the peak abundance of 55.74%, and became the dominant symbiotic flora. Therefore, in the present study, a symbiotic *Serratia* strain was isolated and purified from cultivation bags of *H. marmoreus,* and then identified as *Serratia odorifera* by biochemical testing and 16S rRNA sequencing. At the same time, QS signaling molecules from *S. odorifera* fermentation broth were isolated and purified, and their chemical structures were identified by spectral methods. Furthermore, the effects of these signal molecules on lignin-degrading enzyme activity and related gene expression of *H. marmoreus* were also investigated in detail. The symbiotic effects of the QS system in *S. odorifera* on the growth of hyphae, primordium formation, and fruit body development of *H. marmoreus* need to be explored in depth, which will lay a foundation to shorten production cycles further, reduce the cost of production, and increase the fruit body yield in factory cultivation of *H. marmoreus*.

## 2. Materials and Methods

### 2.1. Microorganism Strains and Chemicals

*H. marmoreus*, *S. odorifera*, *E. coli* S17-1 λ pir, *Chromobacterium violaceum* CV026, and *E. coli* [pSB401] were stored in our laboratory, and all bacteria were cultured at 37 °C in Luria broth (LB) medium composed of 0.5% yeast extract, 1% tryptone, and 1% NaCl. *H. marmoreus* was grown at 25 °C in PDA liquid medium (2% glucose, 0.3% yeast extract, 0.3% tryptone, 0.15% KH_2_PO_4_, 0.15% MgSO_4_·7H_2_O, and 0.01% vitamin B_1_ (VB1) in 1 L of potato infusion). Diketopiperazine standards were purchased from Gil Biochemical Firm (Shanghai, China), and the signaling molecule C_6_-HSL was purchased from Sigma-Aldrich (Mainland China). Other chemicals used in this study were purchased from the Sinopharm Chemical Reagent Co. (Shanghai, China). Restriction enzymes, DNA Ligation Kit Ver. 2.1, RNase, LA Taq Polymerase with GC Buffer and dNTPs were purchased from Bao Bioengineering Co., Ltd. (Dalian, China). Bacterial genome DNA extraction kit, Agarose gel recovery and purification kit, and Bacterial plasmid mini-extraction kit were from Tiangen Biotech Co., Ltd. (Beijing, China).

### 2.2. Evaluation of QS Activity of Crude Extracts by Indicator Strains

The submerged fermentation of *S. odorifera* was conducted in a flask with 400 mL of LB medium on a rotary shaker at 37 °C at 150 rpm. After 24 h of cultivation, the fermentation broth was obtained and centrifuged to remove bacterial cells. The collected supernatant was extracted three times with an equal volume of ethyl acetate and concentrated in vacuo to obtain the crude extracts, which were stored in the refrigerator for further use. Standard disc diffusion assays were performed according to our previous research [27] to detect the QS activity of crude extracts of *S. odorifera* using *C. violaceum* CV026 and *E. coli* [pSB401] as indicator strains [6]. Briefly, when the required indicator strains and kanamycin solution were added to LB solid media, the medium was adequately mixed after the supplements were added and then poured into Petri plates. Then, sterile circular filter papers (6 mm diameter) were taken out, and they absorbed an equal amount of sample solutions with different concentrations of metabolites. C_6_-HSL solution was used as a positive control, and methanol was used as a negative control. The filter paper sheets laid flat on solid media in plates at three different points. They were cultured in an incubator at 37 °C for 24 h. The activated indicator strain *E. coli* [pSB401] and tetracycline solution were also added to LB liquid media containing C_6_-HSL solution, methanol solution, and sample solution, respectively. The flasks were incubated in a shaker at 37 °C and 150 revolutions per minute (rpm) for 24 h. Then, 200 μL of culture solution was taken from each flask, added to a black 96-well plate, and subjected to a multi-purpose microplate reader (SpectraMax M2*^e^*, Molecular Devices, Sunnyvale, CA, USA).

### 2.3. Purification and Identification of QS Signal Molecules from Crude Extracts

Crude extracts were dissolved in dimethyl sulfoxide (DMSO) and sterilized by filtration through a 0.22-micron membrane filter. The preliminary separation was performed by preparative high-performance liquid chromatography (PHPLC) using methanol and ultrapure water as mobile phases in a Waters 1525 system (Waters, Milford, MA, USA). The separated solution was treated with a low-temperature rotary evaporator to remove methanol solvent, and water was removed by a low-temperature freeze dryer. The obtained compounds were then redissolved in methanol for further analysis. The sample solution was sequentially separated by high-performance liquid chromatography (HPLC) in a Waters 2695 system (Waters, Milford, MA, USA) with methanol and ultrapure water (35:65) as the mobile phase by setting different mobile phase gradients (20:80 to 55:45), elution times (50 min), and other parameters (sample injection 10 μL; temperature 25 ℃; flow rate 1 mL/min; wavelength 210 nm) to obtain the optimal separation. *C. violaceum* CV026 and *E. coli* [pSB401] were used as QS indicator strains to track the QS activity of each sample solution. Then, all active components were continuously separated by HPLC until a single peak appeared, and the highly purified signal molecules were obtained for chemical structural analysis.

Purified compounds were placed in silica gel overnight and dried thoroughly, redissolved in deuterated chloroform solution, transferred to a tube, and scanned by nuclear magnetic resonance (NMR) on a Bruker 500 MHz [28]. Signal molecules isolated from *S. odorifera* fermentation broth were detected by ultra high-performance liquid phase-mass spectra (UHPLC-MS) and HPLC as follows: C_18_ reversed-phase column (ZORBAX SB-Aq, 4.6 × 150 mm, 5 μm), methanol and ultrapure water from (20:80) to (55:45), gradient elution for 40 min, injection 10 μL, temperature 25 °C, and flow rate 1 mL/min. MS experiments were carried out using an Agilent 1290/6430 instrument (Agilent, CA, USA), and the conditions were set as follows: ESI (+) ion source, mass range of scan: m/e 130 to m/e 350, ion source temperature 100 °C, desolvation temperature 300 °C, spray voltage 3.4 kV, cone voltage 30 V, and nitrogen flow 6 L/h. To improve the accuracy of structure determinations, the chemical formula of these compounds was also further confirmed by high resolution of the MS (up to 100,000), which provided the precise mass accuracy. Diketopiperazine standards were dissolved in chromatographic grade methanol to prepare 10 mg/mL mother liquor. They were then diluted 10-fold to reach a final concentration of 1 mg/mL. Subsequently, the *S. odorifera* fermentation broth and diketopiperazine standards were subjected to HPLC analysis. The elution conditions were as follows: methanol in ultrapure water (20%–55%) as eluent; flow rate, 1 mL/min; column temperature, 30 °C; injection volume, 10 µL; detection wavelength, 210 nm; and elution time, 50 min.

### 2.4. Determination of Laccase and Manganese Peroxidase Activity

Laccase activity was determined by monitoring the rate of 5 mM 2,2′-azino-bis(3-ethylbenzthiazoline-6-sulfonic acid) (ABTS) oxidizing to its cation radical (ABTS^+^) at 436 nm (*ε_436_* = 29,300 M^−1^ cm^−1^) in 0.1 M sodium acetate buffer (pH 5.0) at 30 °C. The 3 mL reaction system was composed of 2.4 mL of HAc-NaAc buffer solution, 0.2 mL of ABTS solution, and 0.4 mL of crude enzyme. The mixture was preheated in a 30 °C water bath for 5 min, and the changes in absorbance at 436 nm for the first 3 min were recorded after zeroing of the spectrophotometer. One unit (U) of enzyme activity was defined as the amount of enzyme required to oxidize 1 μmol of ABTS per minute at 30 °C. Manganese peroxidase activity was measured spectrophotometrically by monitoring the oxidation of 2,6-Dimethylphenol (2,6-DMP) at 470 nm. The reaction mixture contained 1.6 mL of 0.25 M sodium tartrate buffer solution (pH 3.0), 0.1 mL of 0.5 mM manganese sulfate, 0.1 mL of 0.1 mM hydrogen peroxide, 0.2 mL of 0.2 mM 2,6-DMP solution, and 1 mL of crude enzyme solution, and the mixture was preheated in a 30 °C water bath for 2 min to measure the initial rate of increase in absorbance at 470 nm for the first 3 min. One unit (U) of enzyme activity was defined as the amount of enzyme required to oxidize 1 μmol of 2,6-DMP per minute at 30 °C.

### 2.5. Real-Time Fluorescence Quantitative PCR Detection of Related Gene Expression

The mycelia of *H. marmoreus* were inoculated into PDA liquid medium, and different signal molecules were added to a final concentration of 0.25%. At the same time, control groups with equal amounts of methanol and DMSO were prepared. The 7-day-old mycelia were collected and washed twice with sterilized ultrapure water, immediately frozen in liquid nitrogen, and stored at −80 °C until use for the RNA isolation. The total RNA of *H. marmoreus* mycelia cultured under different conditions was extracted by TRIzol reagent and reverse transcribed to cDNA as a template for quantitative fluorescence reactions. Subsequently, real-time PCRs were performed with cDNA samples distributed in 96-well flat-bottomed plates on an ABI Stepone plus instrument (Applied Biosystems, Foster City, CA, USA). All data analysis was performed by the 2^-ΔΔ^*^CT^* method, which is a convenient way to calculate relative gene expression levels between different samples using the threshold cycles (*CTs*). Furthermore, the 2^-ΔΔ^*^CT^* method was applied to data with automatic removal of background fluorescence by the real-time PCR software.

### 2.6. Construction of NRPS Gene Knockout Strains

A 504 bp fragment of the *NRPS* gene was amplified by PCR from the genomic DNA of *S. odorifera* using a primer pair (*NRPSs1*: AAGGTACCTTGGTCGGTCATCAGGCTATC, *NRPSs2*: AAAAGTACTATTCCACACCGGCAGGCC). Sites of the restriction enzymes *Kpn* I and *Sca* I (underlined regions) were introduced into these two primers to facilitate the follow-up genetic manipulations. Subsequently, this fragment and the suicide vector pUTKm were completely digested with restriction endonucleases *Kpn* I and *Sca* I. The recovered *NRPS* fragments were ligated into the suicide *vector* pUTKm at a molar ratio of 4:1, transformed into *E. coli* S17-1 λpir competent cells by heat shock, and dispensed onto LB agar plates supplemented with kanamycin (100μg/mL). The transformants were selected on LB agar plates containing kanamycin. Then the recombinant plasmid pUTKm-*NRPSs* was extracted and identified by PCR amplification, DNA sequencing, and double restriction enzyme digestion. Next, parental hybridization was performed as follows: *S. odorifera* and *E. coli* S17-1 λ pir (pUTkm-*NRPSs*) strains were cultured in LB broth for approximately 2 h at 37 °C until the optical density of the bacterial culture at 600 nm (OD_600_) reached 0.3–0.5. Next, the fermentation broths of donor strain *E. coli* S17-1 λpir and recipient strain *S. odorifera* were thoroughly mixed at the ratio of 3:1 and centrifuged at 6000 rpm for 5 min to collect bacterial cells, followed by the addition of 100 μL of fresh LB liquid medium. Then, *the bacterial resuspension* was added to the middle point of a filter and incubated for 8–16 h at 37 °C. Bacterial clones grown on the filter were scraped into a centrifuge tube filled with 1 mL of sterile LB liquid media. Finally, 100 μL of the suspension was removed and spread on LB agar plates containing ampicillin (500μg/mL) and kanamycin (100μg/mL) and cultured in an incubator at 37 °C for 16 h.

### 2.7. Comparison of Metabolic Characteristics between S. odorifera and its NRPS Mutant

One milliliter of the 37 °C culture of wild-type *S. odorifera* and its *NRPS* mutant was inoculated into 100 mL of LB liquid medium (1:100 dilution) and then cultivated in a shaker at 37 °C and 150 rpm for 24 h. The fermentation broth was sampled and centrifuged to remove cells completely, and then the supernatant was extracted three times with an equal amount of ethyl acetate to form an organic phase. The ethyl acetate layer was collected, and vacuum concentrated into a paste using a low-temperature rotary evaporator. Next, the concentrate was freeze-dried into a powder, dissolved in methanol (50 mg/mL), and then filtered with a 0.22 μm microporous membrane (Millipore, USA) to ensure the consistency of the samples. Subsequently, these samples were simultaneously analyzed by HPLC, and elution was achieved using a gradient method. The conditions were as follows: C_18_ reversed-phase column (ZORBAX SB-Aq, 4.6 × 150 mm, 5 μm); methanol in ultrapure water (20%–55%) as eluent; flow rate, 1 mL/min; column temperature, 25 °C; injection volume, 10 µL; detection wavelength, 210 nm; and elution time, 50 min.

## 3. Results and Discussion

### 3.1. Activity Evaluation of QS Signal Molecules in S. odorifera

**Figure 1:**
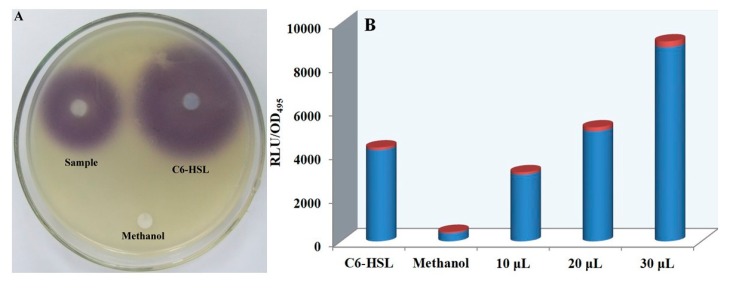
Detection of quorum sensing (QS) signal molecules secreted by *Serratia odorifera* with indicator strains. (**A**) Violacein detection by *Chromobacterium violaceum* CV026; (**B**) measurement of bioluminescence by *Escherichia coli* [pSB401].

*C. violaceum* CV026, a violacein-negative, double mini-Tn5 mutant of *C. violaceum* ATCC 31,532 [29], requires exogenous addition of C_6_-HSL to undergo QS and produce violacein. *E. coli* [pSB401] harbors *V. fischeri luxR* and the promoter region of *luxI* fused to *luxCDABE* from *Photorhabdus luminescens* [30,31]. It responds to exogenous short-chain AHLs, resulting in light emission. Therefore, these engineered bacteria can be used as indicator strains for the activity evaluation of QS signal molecules in *S. odorifera*. Ten microliters of *S. odorifera* extracts were loaded on the filter sheet as testing samples, and different filter sheets loaded with C_6_-HSL solution and methanol were used as positive and negative controls, respectively. The plates were incubated overnight at 28 °C and observed for any growth halos and inhibition zones. The production of violacein was determined spectrophotometrically at a wavelength of 585 nm. The results showed that the violacein was generated around the filter sheets impregnated by *S. odorifera* extract and C_6_-HSL solution (Figure 1A). Compared to that of the negative control group, the bioluminescence intensity induced by *S. odorifera* extracts increased gradually with increasing concentrations of extracts (Figure 1B), which indicated that *S. odorifera* could synthesize and release QS signal molecules.

### 3.2. Isolation and Identification of QS Signal Molecules from S. odorifera

**Figure 2:**
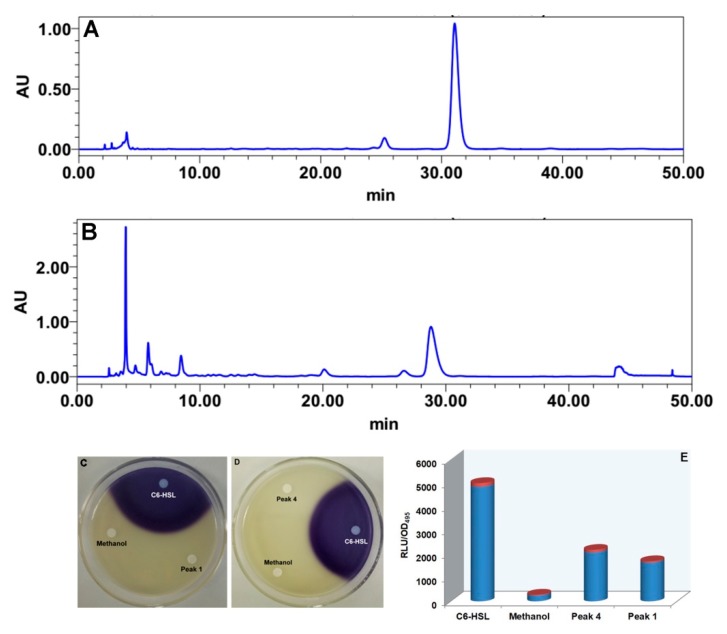
High-performance liquid chromatography (HPLC) analyses of peaks 1 and 4 and their QS activity evaluation. (**A**) Peak 4; (**B**) peak 1; (**C**,**D**) violacein detection by *C. violaceum* CV026; (**E**) measurement of bioluminescence by *E. coli* [pSB401].

**Figure 3:**
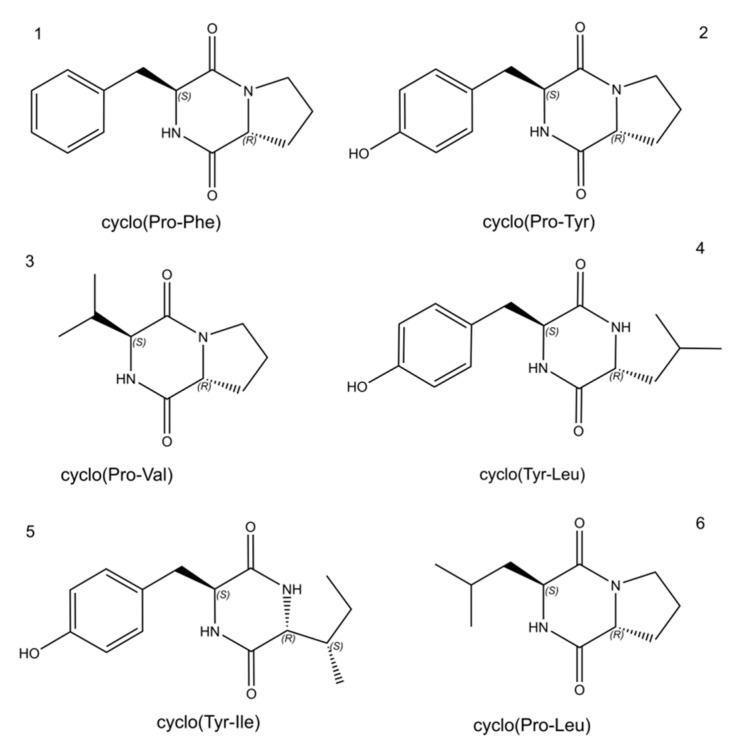
Chemical structures of cyclic dipeptides acting as QS signaling molecules produced by *S. odorifera.*

Four active fractions (A: 4–10 min, B: 10–20 min, C: 20–28 min, D: 28–46 min) were originally isolated from fermentation broth extracts of *S. odorifera* by using repeated silica gel column and Sephadex LH-20-based column chromatography system. Fractions D and A were separated sequentially by HPLC using methanol and ultrapure water as mobile phases under different elution conditions. Fraction D was eluted by a constant gradient with a mobile phase of acetonitrile and ultrapure water *at a ratio of* 10:90 for 50 min. The peak appearing at approximately 30 min was gathered and named peak 4 (Figure 2A). Similarly, fraction A was eluted in constant gradient mode with acetonitrile and ultrapure water *at a ratio of* 5:95 as a mobile phase to obtain a single peak, 1, at 28 min (Figure 2B). The results showed that these two compounds cannot induce the secretion of purple pigment in *C. violaceum* CV026 (Figure 2C,D), but could stimulate bioluminescence emission by *E. coli* [pSB401] (Figure 2E). This absence of violacein synthesis indicated that these two single compounds were not short-chain AHL molecules but other types of signaling molecules responsible for QS activity. As shown in Table S1, the analysis of compound 4 was carried out by NMR spectroscopy to determine its chemical structure. A proton spectrum (Figure S1) showed that a single peak was an NH group at 5.59 ppm. The signals from 1.91 to 4.29 ppm were considered to represent CH2 and CH groups, and the signal from 7.22 to 7.36 ppm was assigned to aromatic hydrogen atoms. A carbon spectrum (Figure S2) indicated that the compound contained a total of 14 carbon atoms and showed two amide carbonyl signals, one at 165.05 and one at 169.37 ppm. The ^1^H-detected heteronuclear multiple bond correlation data showed that H-3 and C-4, H-4 and C-5, H-5 and C-4, H-5 and C-7, H-6 and C-5, and H-6 and C-7 and C-3 (45.47 ppm) were chemically related, giving R_2_N-CH_2_-CH_2_-CH_2_-CHR-CONR_2_. H-9 and C-1 and C-10; H-10 and C-1, C-1’, and C-2’; H-2’ and C-10 and C-4’; H-3’ and C-1′; and H-4′ and C-2′ had CH covalent bond-related signals, which indicated that the fragment Ar-CH_2_-CHR-CO-NR_2_ was also present in this compound. NMR spectroscopy analysis showed that this compound was cyclo(Pro-Phe) (1) (Figure 3 1), which was consistent with the literature data [32].

As shown in Table S2, the analysis of the molecular structure of compound 1 was carried out by NMR spectroscopy. The proton spectrum (Figure S3) showed two signals in the low-field region (δ_H_ 9.17, s, 4’-OH; δ_H_ 7.85, s, 8-NH) and four benzene ring protons in the aromatic zone (δ_H_ 7.04, d, H-2’; δ_H_ 6.62, d, H-3’; δ_H_ 6.62, d, H-5’; δ_H_ 7.04, d, H-6’). Absorption peaks overlapped and were split as the d peak, which was presumed to reflect an internally 1,4-disubstituted benzene ring structure. Four groups of fatty protons signals (δ_H_ 2.92, 2.00, 1.71, 1.40) and four groups of heteroatomic aliphatic proton signals (δ_H_ 4.23, 4.03, 3.40, 3.25) were found in the high-field ^1^H Magic-Angle Spinning Nuclear Magnetic Resonance spectra (^1^H MAS NMR), which is a technique for the quantitative determination of hydrogen types. In particular, two proton signals, one at 4.23 (1H, t, J = 4.6 Hz) and one at 4.03 (1H, t, J = 9.2 Hz), were due to structural features of a cyclic dipeptide. The carbon spectrum (Figure S4) showed that this compound contained 14 carbon atoms with two amide carbon signals (δ_C_ 165.54; 169.35), six benzene ring carbon signals (δ_C_ 127.51; 131.25; 115.22; 156.34; 115.22; 131.25), three signals for carbon with attached heteroatoms (δ_C_ 44.99; 58.84; 56.46) and three saturated aliphatic carbon signals (δ_C_ 22.30; 28.28; 35.16). According to the proton and carbon spectra, it was speculated that this compound was cyclo(Pro-Tyr) (2) (Figure 3 2), which coincided with the published literature [32].

The crude extracts of *S. odorifera* fermentation broth were also analyzed by UPLC-MS. A summary of the UPLC-MS data and identification of the compounds in the extracts of *S. odorifera* are presented in Figure S5. The molecular weight and chemical formula of the target peak compound were compared with those of database entries. The major UPLC-MS peaks of molecular ion with different *m*/*z* ratios from extracts of *S. odorifera* yielded six compounds, which were identified as cyclic dipeptides based on the characteristic fragmentation pattern. The results showed that the two short peptides with *m*/*z* ratios (245.1286 and 261.1236) and retention times (approximately 2.464 and 6.241 min) were cyclo(Pro-Phe) and cyclo(Pro-Tyr) (Figure 3 1/2), respectively. The bioactive metabolites in the fermentation broth of *S. odorifera* still contained four cyclic dipeptides (Figure 3 3/4/5/6): cyclo(Pro-Val) at retention time 2.795 min with *m*/*z* ratio of 197.1284, cyclo(Tyr-Leu) at retention time 4.08 min with *m*/*z* ratio of 277.1549, cyclo(Tyr-Ile) at retention time 4.750 min with *m*/*z* ratio of 277.155, and cyclo(Pro-Leu) at retention time 5.612 min with *m*/*z* ratio of 211.1443. These compounds are diketopiperazine-like signal molecules and were simultaneously also verified with standard diketopiperazine signal molecules by HPLC (Figure S6). In order to understand better how to interact with enzymes in subsequent research, the tridimensional structures of all diketopiperazines were predicted and simulated using AutoDockVina (data not shown).

### 3.3. Effects of Signal Molecules on the Activity of Lignin-Degrading Enzymes

**Figure 4:**
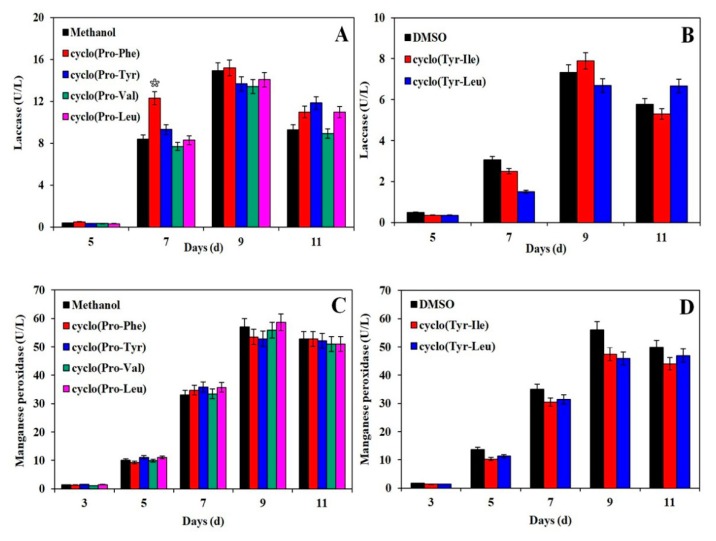
Effects of signal molecules on the activity of lignin-degrading enzymes secreted by *Hypsizygus marmoreus.* (**A**,**B**) Effects of signal molecules dissolved in methanol and dimethyl sulfoxide (DMSO) on laccase activity; (**C**,**D**) effects of signal molecules dissolved in methanol and DMSO on manganese peroxidase activity. All measurements in these experiments were made in triplicate. Bars are significantly (*p* < 0.05) different from each other by T-test.

Extracellularly secreted enzymes of fungal mycelia can accelerate the degradation of substrates used in edible fungus cultivation. Therefore, the effects of cyclo(Pro-Phe) (1) and cyclo(Pro-Tyr) (2) on extracellular enzymes were investigated in the cultivation of *H. marmoreus*. As shown in Figure 4A, the addition of cyclo(Pro-Phe) (1) promoted the laccase secretion significantly. The laccase activity increased 1.32-fold as compared with the control group on the 7th day. The significant analysis was performed at *p* < 0.05 level by T-test, which showed that the difference among the average values of different groups reached the significant level. The laccase activity could reach a maximum value of 15.19 U/L on the 9th day. When the cyclo(Pro-Tyr) was added to the culture substrate, the laccase activity increased 15.15% and reached 10.96 U/L. The laccase activity was also enhanced by the addition of cyclo(Tyr-Ile) (5) to cultivation substrates, reaching 7.889 U/L on the 9th day. With the cyclo(Tyr-Leu) (4) feeding strategy, the laccase activity increased continuously, reaching a value 1.2-fold higher than that of the control groups on the 11th day (Figure 4B). Similarly, manganese peroxidase secretion was also improved by the addition of cyclo(Pro-Phe) (1) and cyclo(Pro-Tyr) (2) to cultivation substrates, and manganese peroxidase activity was 1.2 and 1.1 times higher in groups with these supplements than in control groups on the 7th day, respectively. The statistical analysis showed that the changes of manganese peroxidase activity had no significant difference at 5% level among the average values of different groups, compared to the control groups (Figure 4C). Cyclo(Tyr-Leu) (4) and cyclo(Tyr-Ile) (5) had no positive effect on the secretion of manganese peroxidase (Figure 4D).

### 3.4. Quantitative RT-PCR Analysis of Gene Expression

**Figure 5:**
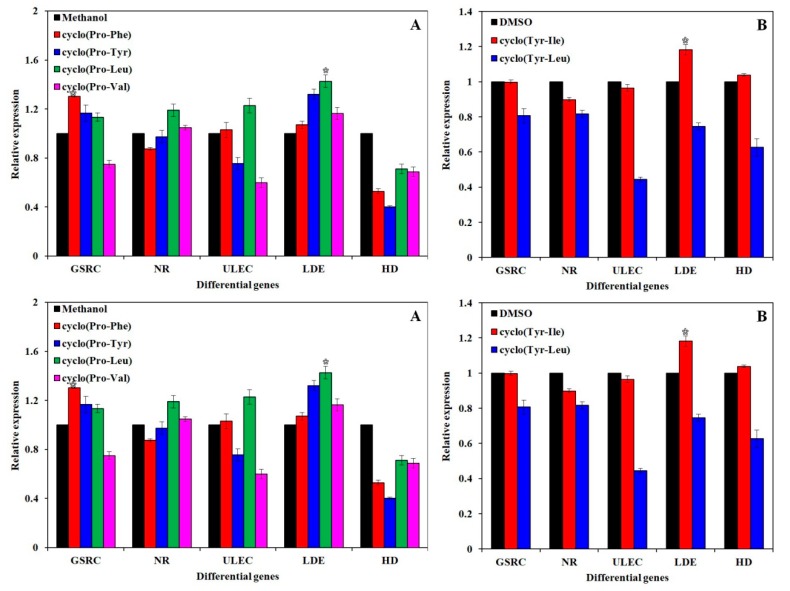
Effects of signal molecules dissolved in methanol and DMSO on the expression of differential genes (Table 1) in the growth and development of *H. marmoreus*. (**A**) Effects of cyclo(Pro-Phe) (1), cyclo(Pro-Tyr) (2), cyclo(Pro-Val) (3), and cyclo(Pro-Leu) (6) dissolved in methanol on the expression of differential genes; (**B**) effects of cyclo(Tyr-Leu) (4) and cyclo(Tyr-Ile) (5) dissolved in DMSO on the expression of differential genes. All experiments were carried out in triplicate. Bars are significantly (*p* < 0.05) different from each other by T-test.

The relative expression of five differentially expressed genes [33] (Table 1) involved in the growth and development of *H. marmoreus* was analyzed by the 2^-^^△△CT^ method under different conditions with the addition of different signal molecules. As shown in Figure 5, the addition of cyclo(Pro-Leu) (4), cyclo(Pro-Tyr) (2), and cyclo(Pro-Phe) (1) facilitated the transcription of SNAP receptor complex member 1. The signal molecules other than cyclo(Tyr-Leu) (4) significantly increased the transcription level of lignin-degrading enzyme genes compared to that in the control groups. Cyclo(Pro-Leu) (6) significantly increased the transcription of four differentially expressed genes while inhibiting the transcription of the 3-hydroxyisobutyrate dehydrogenase gene. Cyclo(Pro-Leu) (6) can also accelerate the lignin degradation, improve the transcription of the NADH reductase gene, and promote oxidative phosphorylation in energy production. Different types of signaling molecules certainly play different roles in regulating the growth and development of *H. marmoreus*.

### 3.5. NRPS Gene Knockout and Construction of Deletion Mutants of S. odorifera

In general, the biosynthesis of cyclic dipeptide in bacteria was mainly catalyzed by the NRPS. Therefore, to verify the synthesis mechanism of six cyclic dipeptides, the function of *NRPS* gene was firstly chosen and studied. An *NRPS* gene fragment of approximately 504 bp was obtained by PCR from the genomic DNA of *S. odorifera* using the primers *NRPSs1* and *NRPSs2* (Figure S7). The resulting PCR product was cloned into the plasmid pUTkm to create pUTkm-*NRPSs*. The identity of the PCR product was verified by PCR and dideoxynucleotide sequencing using the extracted recombinant plasmid as the template. The results showed that the recombinant plasmid contained a partial *NRPS* gene fragment, which was 95% homologous to the corresponding fragment of the original sequence (Figure S8), indicating that the recombinant suicide vector was constructed successfully. Marker exchange mutagenesis with pUTKm-*NRPSs* was achieved using a protocol similar to the report [29]. Transconjugants were selected on NB medium containing 500 µg/mL ampicillin and 100 µg/mL kanamycin. To confirm whether the *NRPS* gene had been inserted into the genome of *S. odorifera*, the primer pair of *NRPSs3* (ATCGTCACTAATCCGGCACAGC) and *NRPSs4* (GAAGTAAGTTGGCCGCAGTG) was designed based on the upstream sequences of the *NRPS* gene and the plasmid vector. The correct disruption of the *NRPS* gene in the genome of *S. odorifera* was confirmed by PCR analysis and dideoxynucleotide sequencing, thereby generating a fragment of approximately 780 bp from genomic DNA *of* the *S. odorifera* mutant (Figure S9). The sequencing results also showed that the sequence of the PCR-amplified product was completely consistent with the original sequence, which indicated that the exogenous plasmid pUTkm was indeed inserted into the *NRPS* gene.

### 3.6. Comparison of QS Signal Molecule Synthesis between Wild-Type S. odorifera and Its Mutant

**Figure 6:**
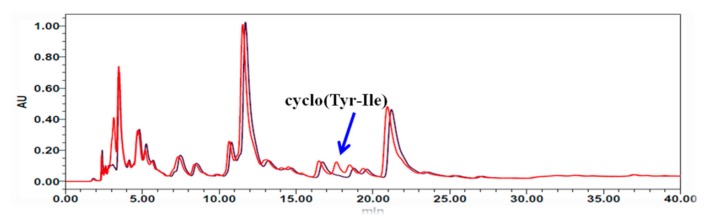
Comparison of QS signaling molecule synthesis between a wild-type *S. odorifera* strain and its mutant. Wild-type *S. odorifera* (red); *S. odorifera* mutant (black).

*Fermentation* broth extracts of *S. odorifera* and its *NRPS* mutant were treated separately and then subjected to HPLC analysis. As shown in Figure 6, the production of cyclo(Tyr-Ile) (5) by the *S. odorifera* mutant was significantly lower than that by wild-type *S. odorifera*. However, the contents of other diketopiperazine-type signaling molecules in the mutant were almost equal to those of the wild-type *S. odorifera* strain. Therefore, it is preliminarily concluded that NRPS catalyzed the synthesis of QS signal molecules in *S. odorifera* [34]. However, there is another biosynthesis pathway for cyclic dipeptide in bacteria, which is catalyzed by the cyclodipeptide synthase (CDPS) [35]. The biosynthesis of QS signal molecules may also be regulated by CDPS, which requires further studies of the synthesis pathways of six cyclic dipeptides by knocking out the CDPS gene from *S. odorifera* to verify this biosynthesis mechanism.

## 4. Conclusions

In summary, diketopiperazine-type signal molecules were successfully isolated and identified from crude extracts of *S. odorifera* fermentation broth using biological and chemical methods. These signal molecules can effectively enhance the transcriptional level of related genes and the activity of lignin-degrading enzymes. At the same time, it was found that the content of cyclo(Tyr-Ile) (5) was decreased and the levels of other diketopiperazine-type signal molecules remained essentially unchanged when the *NRPS* gene was knocked out. Therefore, it is concluded that the *NRPS* gene regulates the synthesis of some QS signal molecules in *S. odorifera*. The mechanism by which the QS system in *S. odorifera* promotes the growth and development of *H. marmoreus* needs to be further explored after the deletion of the *CDPS* gene, which will also lay the foundation for analyzing the synthesis pathway and function of bacterial QS signal molecules.

## Figures and Tables

**Table 1:** Significantly upregulated genes.

NO	Gene_id	Name	Description
**1**	Cluster-6297.30302	Golgi SNAP Receptor Complex 1 (GSRC)	SNARE protein regulating membrane transport
**2**	Cluster-6297.52145	NAPH reductase (NR)	Oxidative phosphorylation
**3**	Cluster-6297.30918	Ubiquitin-protein ligase E3 C (ULEC)	Ubiquitin-mediated proteolysis
**4**	Cluster-6297.28514	Lignin-degrading enzyme (LDE)	Related to lignin degradation
**5**	Cluster-6297.32940	3-Hydroxyisobutyrate dehydrogenase (HD)	Proline, leucine and isoleucine degradation

## Supplementary Materials

The following are available online at https://www.mdpi.com/2218-273X/10/2/298/s1, Figure S1: ^1^H NMR spectra (500 MHz) of compound 4 dissolved in deuterochloroform, Figure S2: ^13^C NMR spectra (500 MHz) of compound 4 dissolved in deuterochloroform, Figure S3: ^1^H NMR spectra (500 MHz) of compound 1 dissolved in deuterochloroform, Figure S4: ^13^C NMR spectra (500 MHz) of compound 1 dissolved in deuterochloroform, Figure S5: UPLC-MS spectra of fermentation broth extracts and identification of each compound, Figure S6: HPLC analysis of signaling molecules, Figure S7: Agarose electrophoresis of the PCR-amplified NRPSs fragment, Figure S8: Agarose electrophoresis of PCR-amplified NRPSs fragment from the recombinant plasmid, Figure S9: The identification of mutant using PCR, Table S1: NMR data of compound 4, Table S2: NMR data of compound 1.
