# Supplementary material for: Cyclic Dipeptides Mediating Quorum Sensing and Their Biological Effects in Hypsizygus Marmoreus
Source: Biomolecules. 2020 Feb 13;10(2):298. doi: 10.3390/biom10020298 (PMC7072446; doi:10.3390/biom10020298)
Supplement: Supplementary file 1 [file biomolecules-10-00298-s001.pdf]

Supporting Information

Cyclic Dipeptides Mediating Quorum Sensing and Their Biological Effects in *Hypsizygus Marmoreus*

Shu-Jing Sun [1,*,†,], Yun-Chao Liu [1,†], Cai-Hong Weng [2,†], Shi-Wei Sun [2], Fan Li [1], Hui Li [2] and Hu Zhu [3,*]

[1] College of Life Sciences, Fujian Agriculture and Forestry University, Fuzhou 350002, People's Republic of China; yunfng0033@126.com (Y.-C.L.); fanli2020@126.com (F.L.)

[2] Centre for Bioengineering and Biotechnology, China University of Petroleum (East China), 66 Changjiang West Road, Qingdao 266580, People's Republic of China; chweng@ipe.ac.cn (C.-H.W.); sunshiwei_1986@163.com (S.-W.S.); lihui@upc.edu.cn (H.L.)

[3] Fujian Provincial University Engineering Research Center of Industrial Biocatalysis, College of Chemistry and Materials Science, Fujian Normal University, 32 Shangsan Road, Fuzhou 350007, People's Republic of China

* Correspondence: shjsun2004@126.com (S.-J.S.); zhuhu@fjnu.edu.cn (H.Z.); Tel.: +86-591-83-789-492 (S.-J.S.); +86-591-83-465-326 (H.Z.)

Short title: Cyclic dipeptides and their functions.

Table S1. NMR data of compound 4.

Carbon Atom Number	δ_C	δ_H
1	165.05	
3	45.47	3.58-3.62(2H, m)
4	22.56	1.91-2.04(2H, m)
5	28.36	2.01-2.04(1H, m)<br>2.33-2.35(1H, m)
6	59.14	4.09(1H, t)
7	169.37	
NH		5.59(s)
9	56.18	4.28(1H, dd)
10	36.79	2.78(1H, dd)<br>3.62-3.66(1H, m)
1′	135.93	7.22-7.36(5H, m)
2′	129.29	
3′	129.10	
4′	127.57	

Table S2. NMR data of compound 1.

Carbon Atom Number	δ_C	δ_H
1	165.54	
3	44.99	3.25m<br>2.93m
4	22.30	1.70m
5	28.28	2.0m<br>1.40m
6	58.84	4.03t
7	169.35	
9	56.46	4.23t
10	35.16	3.40m<br>2.93m
1′	127.51	
2′	131.25	7.04d
3′	115.22	6.62d
4′	156.34	
5′	115.22	6.62d
6′	131.25	7.04d
4′ -OH		9.17
8-NH		7.85

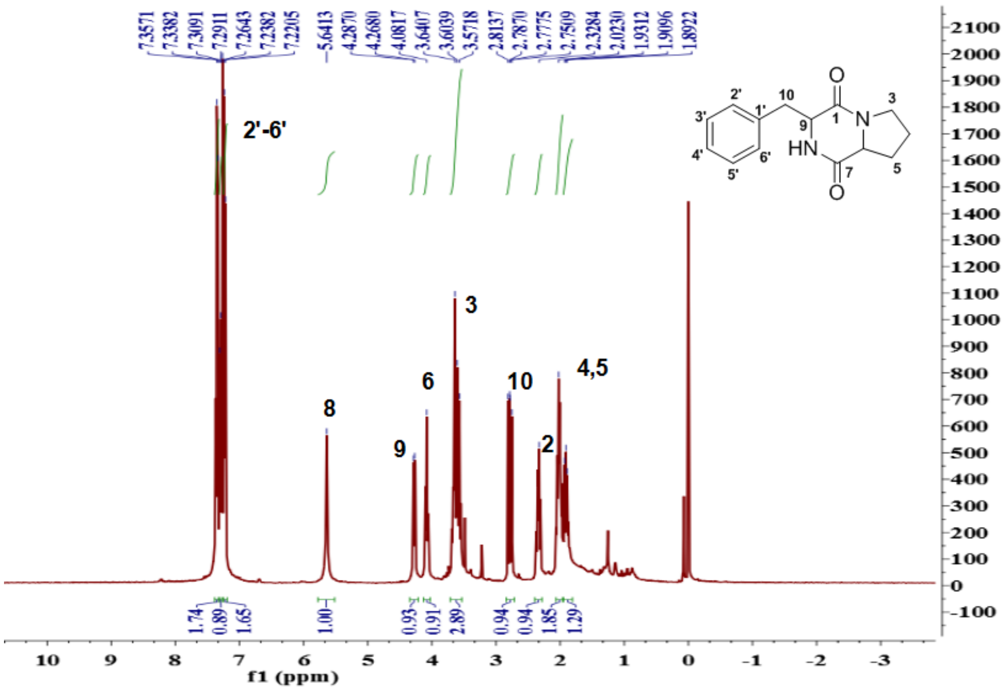

Figure S1. ^{1}H NMR spectra of compound 4.

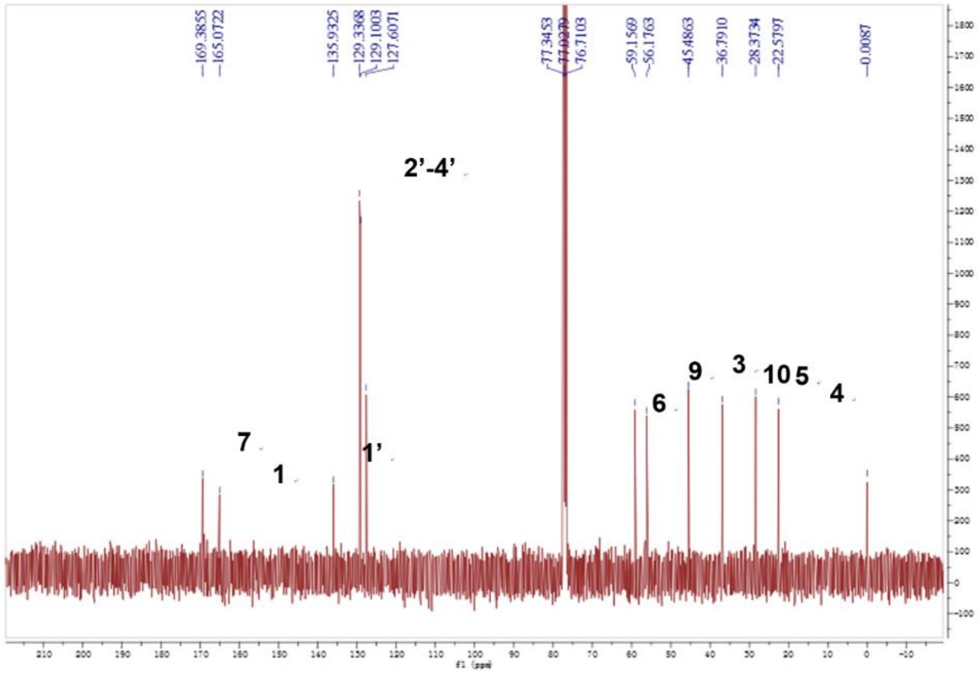

Figure S2. ^{13}C NMR spectra of compound 4.

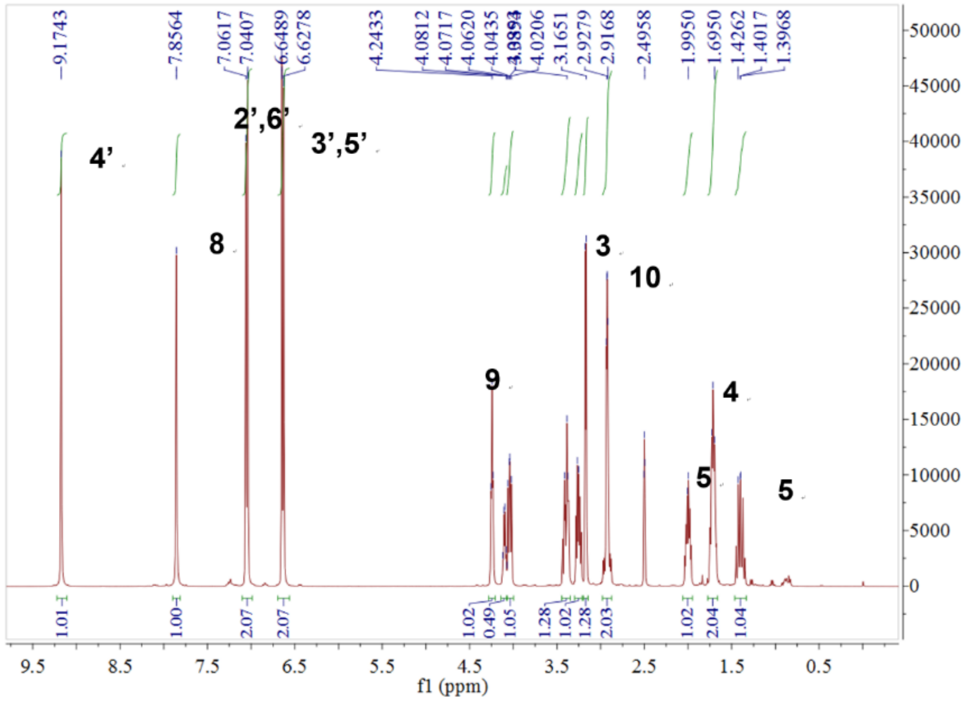

Figure S3. ^{1}H NMR spectra of compound 1.

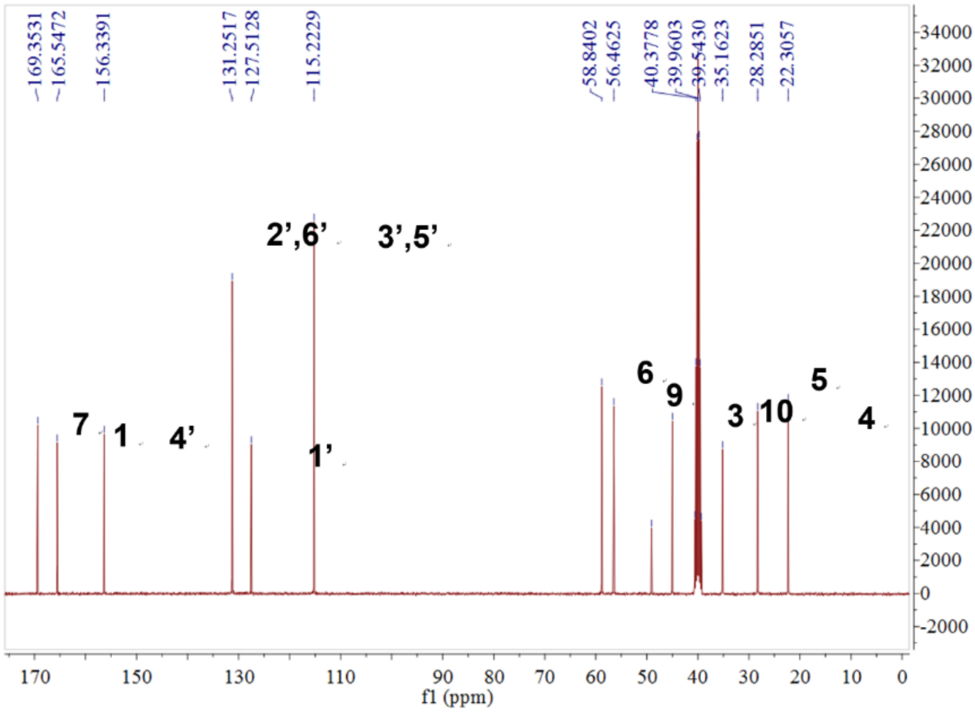

Figure S4. ^{13}C NMR spectra of compound 1.

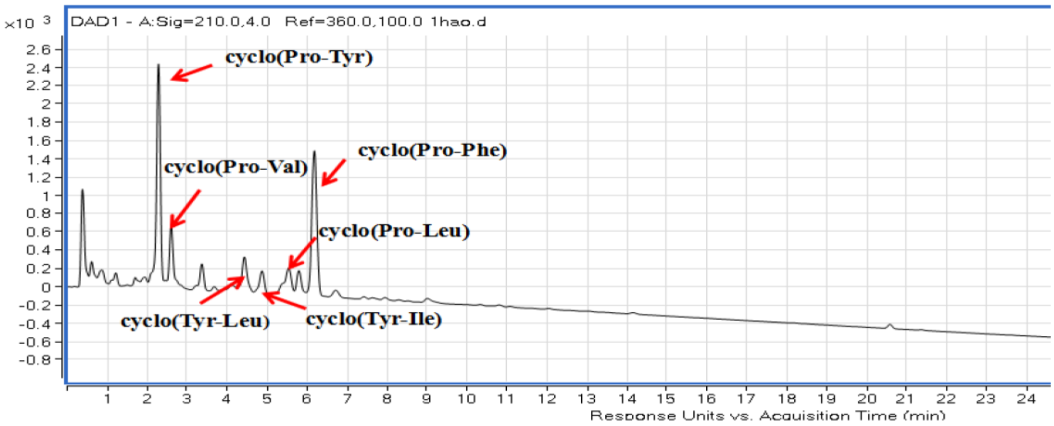
DAD1 - A:Sig=210.0,4.0 Ref=360.0,100.0 1hao.d
x10 3
cyclo(Pro-Tyr)
cyclo(Pro-Phe)
cyclo(Pro-Val)
cyclo(Pro-Leu)
cyclo(Tyr-Leu)
cyclo(Tyr-Ile)
Response Units vs. Acquisition Time (min)

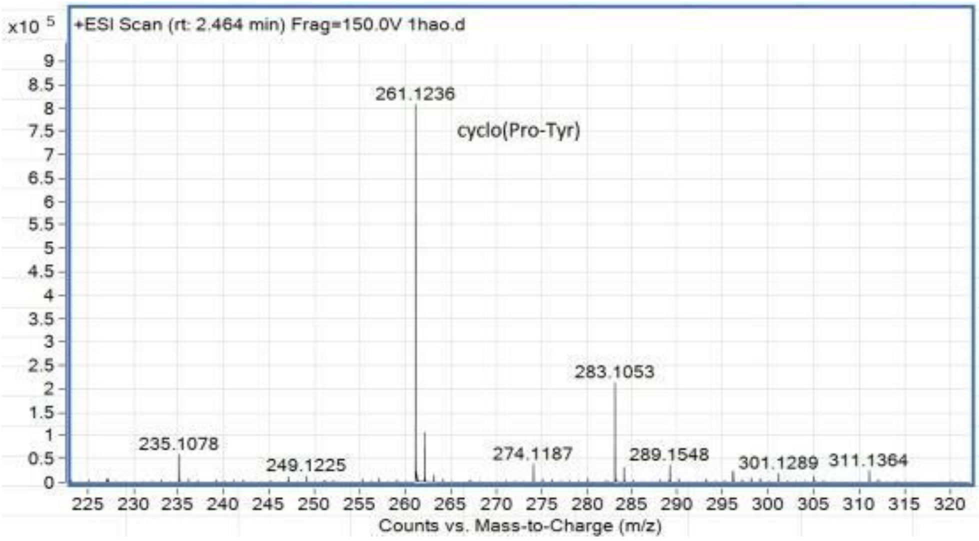
+ESI Scan (rt: 2.464 min) Frag=150.0V 1hao.d
x10 5
261.1236
cyclo(Pro-Tyr)
283.1053
235.1078
249.1225
274.1187
289.1548
301.1289
311.1364
Counts vs. Mass-to-Charge (m/z)

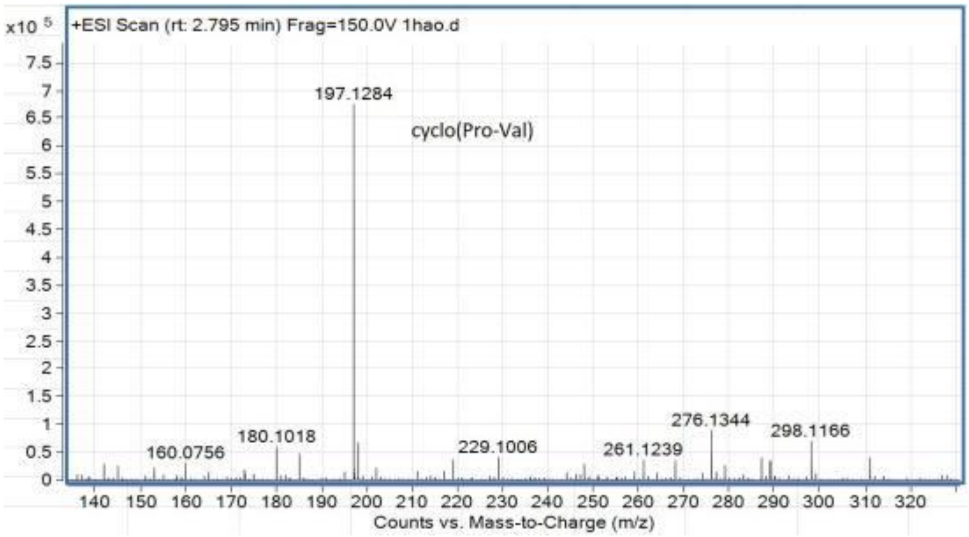
+ESI Scan (rt: 2.795 min) Frag=150.0V 1hao.d
x10 5
197.1284
cyclo(Pro-Val)
276.1344
298.1166
180.1018
160.0756
229.1006
261.1239
Counts vs. Mass-to-Charge (m/z)

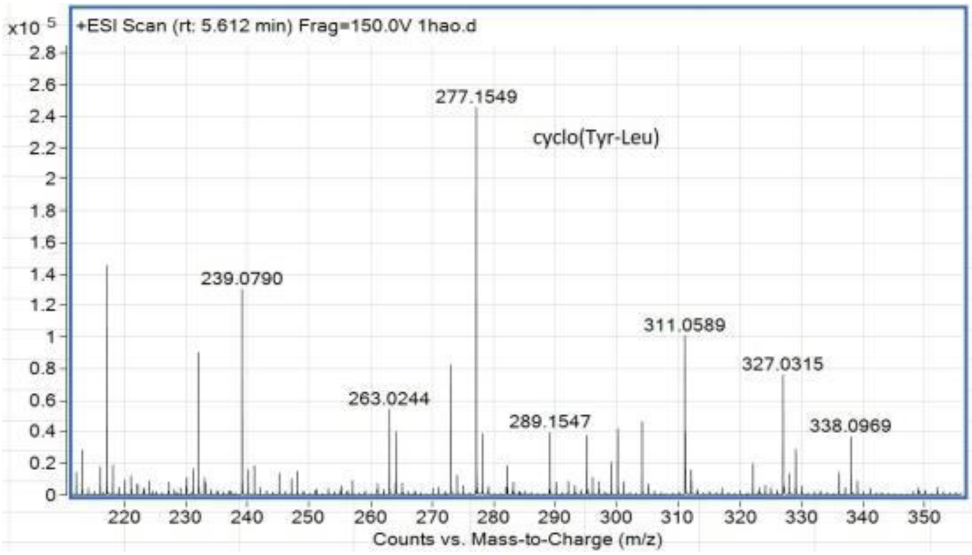
x10 5
+ESI Scan (rt: 5.612 min) Frag=150.0V 1hao.d
277.1549
cyclo(Tyr-Leu)
239.0790
311.0589
327.0315
263.0244
289.1547
338.0969
Counts vs. Mass-to-Charge (m/z)

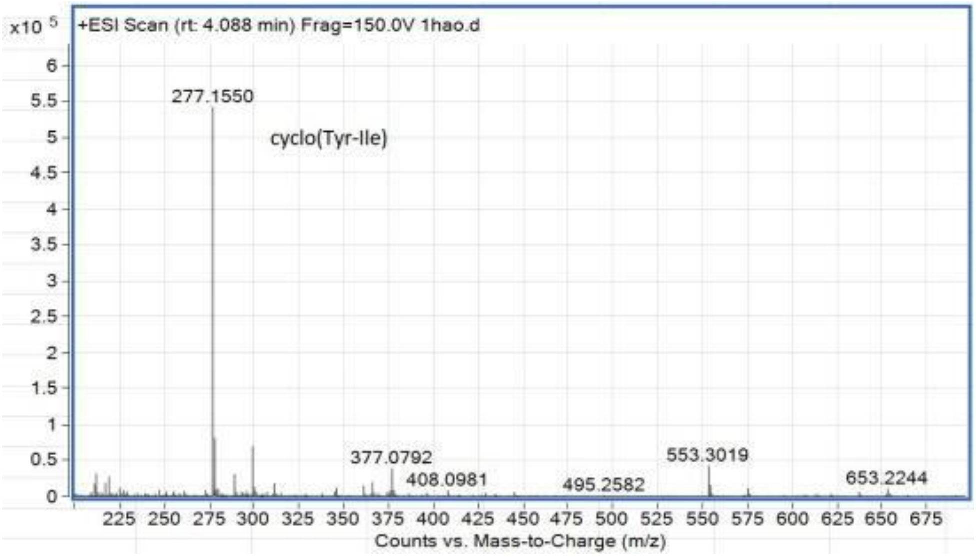
x10 5
+ESI Scan (rt: 4.088 min) Frag=150.0V 1hao.d
277.1550
cyclo(Tyr-Ile)
377.0792
408.0981
495.2582
553.3019
653.2244
Counts vs. Mass-to-Charge (m/z)

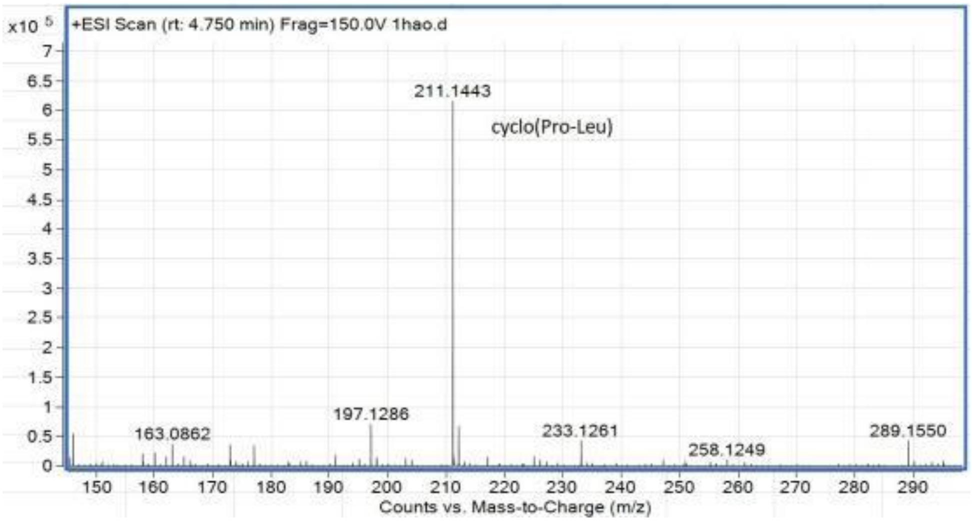
x10 5
+ESI Scan (rt: 4.750 min) Frag=150.0V 1hao.d
211.1443
cyclo(Pro-Leu)
197.1286
163.0862
233.1261
258.1249
289.1550
Counts vs. Mass-to-Charge (m/z)

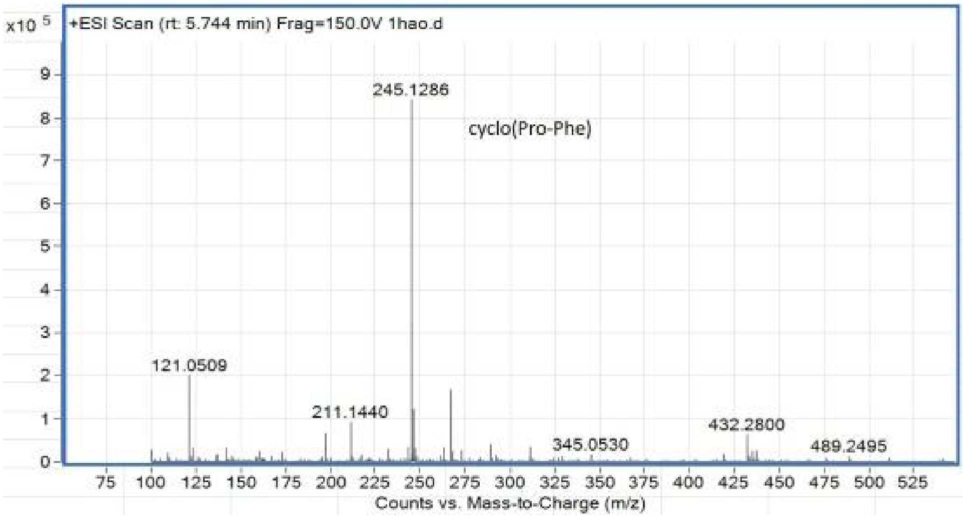

Figure S5. UPLC-MS spectra of fermentation broth extractsandidentification of each compound.

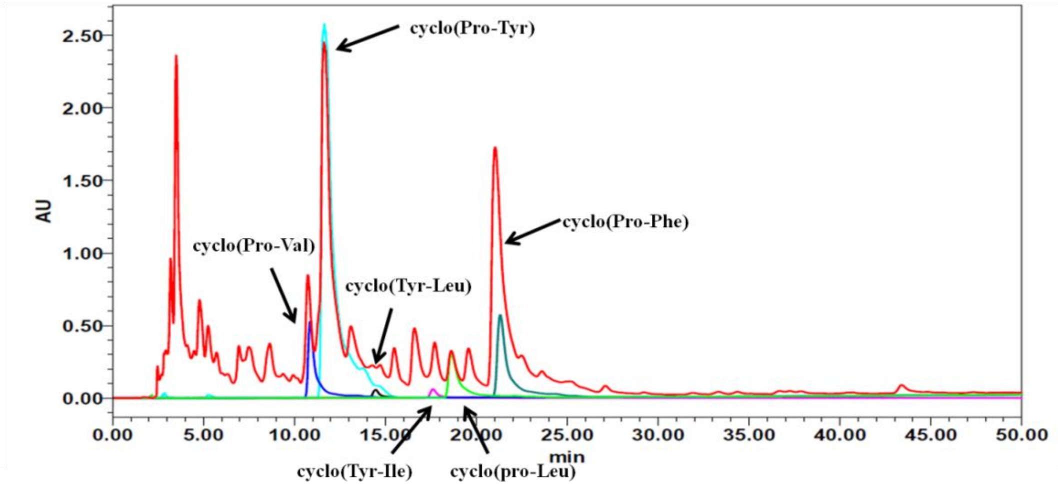

Figure S6. HPLC analysis of signaling molecules.

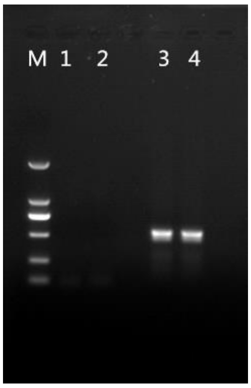

Figure S7. Agarose electrophoresis of the PCR-amplified NRPSs fragment. M: DNA marker DL2000; 1-2: blank control; 3-4: partial NRPSs gene fragment.

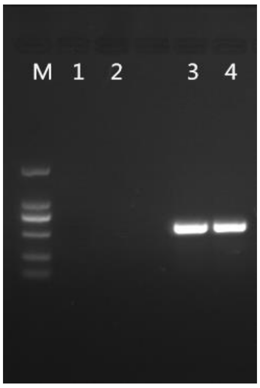

Figure S8. Agarose electrophoresis of PCR-amplified *NRPSs* fragment from the recombinant plasmid. M: DNA marker DL2000; 1-2: blank control; 3-4: partial *NRPSs* gene fragment.

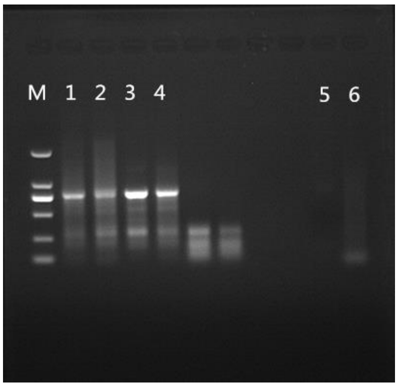

Figure S9. The identification of mutant using PCR. M: DNA marker DL2000; 1–4: Amplification of *NRPSs* fragment using genomic DNA of *Serratia odorifera* as template; 5–6: Amplification of *NRPSs* fragment using genomic DNA *of Serratia odorifera* mutant as template.
